# Tooth-Caries of Pregnancy—Its Cause and Treatment

**Published:** 1880-04

**Authors:** Edward C. Kirk

**Affiliations:** Philadelphia.


### ARTICLE V.

### *Tooth-Caries of Pregnancy—Its Cause and Treatment.*

BY EDWARD C. KIRK, D. D. S., PHILADELPHIA.

It is a well-known fact that during pregnancy women are often subject to more or less annoyance and discomfort from their teeth. This disturbance may vary in degree, from a slight uneasiness—a mere consciousness on the part of the individual of the presence of teeth in her mouth—to the severest form of odontalgia, involving several teeth. The frequent occurrence of rapid and extensive destruction of tooth-structure during pregnancy is so well recognized that it would be useless to multiply examples; one case will serve to illustrate. Mrs. J. presented herself for examination of her teeth. She gave the following history. Up to the time of her marriage the teeth had been of good quality, rarely requiring the services of a dentist. During the three years following marriage she gave birth to two children; she also suffered much from toothache, and had two of her teeth extracted. After the birth of her second child she placed herself under the care of a dentist in a neighboring city, who put her mouth in order; he being a thorough and conscientious operator, the work was well done.

Again she became pregnant, suffering as before with her teeth; some of the fillings dropped out, and a number of new cavities appeared. It was after the birth of this her third child that she came under my charge. On examination, I found the teeth very sensitive, and so soft that they could be cut away almost like chalk. The decayed portions were of that peculiar cartilaginous character which indicates a loss of the mineral portions of the tooth. I filled, in all, seventeen cavities, some large, others small, and extracted the root of one tooth too far gone to be of service in sustaining an artificial crown.

This case is a typical one, and illustrates well a class of cases that call for a large share of the dentist's attention. In those cases where women have borne children rapidly, it is the common story that up to the time of marriage the teeth were of good quality and gave but little trouble, but since have rapidly failed.

As to the cause of this degeneration of tooth structure during pregnancy, there is little reason to doubt the accepted explanation that an excessive demand is made upon the system of the mother for the lime-salts necessary to the formation of the osseous structures of the fœtus, and the teeth of the mother suffer, along with her osseous system, in meeting this demand when the supply of lime salts is not sufficiently kept up in the mother's food.

We believe that much can be done to avert this wholesale destruction of the teeth, the loss of which entails so much disfigurement and physical suffering. If the cause be as stated, then to supply food rich in lime combinations is the rational indication. But most of the food brought to our tables is not rich in bone-forming material, and it may be that even a liberal supply of lime-containing food would not meet the urgent demands made during pregnancy upon a system already poor in lime salts; certainly the judicious use of some of the soluble preparations of lime, such as the lactophosphate or hypophosphate, would be of benefit in such a case, not only in maintaining the lime standard of the mother, but also in insuring to the fœtus a well developed osseous and dental organization. We have every reason to believe that rickets is due to lime-starvation on the part of the mother and child; and evidence is not wanting to show that certain malformations of the jaws, and consequent irregularities of the teeth, are in a measure due to the lack of sufficient bone-forming material during fœtal development.

A fact in this connection which I have had occasion to observe more than once is that in a large number of pregnant women the morbid craving, so called, for unusual articles of food—which is so often present and may occasion great annoyance to both patient and physician—is for articles of a mineral character, as chalk, slate-pencils, lime, plater, whiting, etc.

Two cases in particular have come under my notice. In the first case the woman would mix a saucerful of whiting and water, which she kept near her during the day, and would eat large quantities of it with evident relish.

In the other case the lady stated that during her pregnancies her desire for lime was so great that she would almost have to run past a mortar bed in the street lest the desire to stop and eat portions of it should overcome her. When at home, she would pick particles of plaster and whitewash from the wall and eat them greedily.

It seems reasonable to believe that this craving is nothing more than nature's method of expressing the need for lime-salts when, from pregnancy or other causes, the supply is not equal to the demand and the system is poor in lime as a consequence. I say from other causes; for what else is it that will make a rapidly-growing, overworked school-girl chew her slate-pencils and lead-pencils with such apparent relish?

If all this be true, then the treatment before indicated of supplying to the system all the lime it needs, either by properly selected food, or, if occasion demands it, by the administration of a sufficient quantity of some soluble preparation of lime, ought to do much towards averting the destruction of the teeth by caries during pregnancy, and relieve the distressing cravings for unusual kinds of food incident to that period. As having bearing upon the subject and showing that an increased amount of lime is demanded by the system during pregnancy, I may cite the fondness which birds and fowls generally have for lime, oyster shells, plaster, etc., during the egg-laying period. Another point which I have noted is that this fondness for lime is displayed on the part of the female more than on that of the male; hens will quarrel for the possession of an empty egg-shell, and the cock will look on without interest while they devour it greedily.

The effect of an insufficieat supply of lime is seen sometimes in the case of caged birds, as canaries. If they are not supplied with cuttle-fish bone during the breeding-season, the eggs will be laid without shells, or with shells so thin that they will not withstand the slightest touch. This same result takes place with hens that are cooped for a length of time and are not supplied with a proper quantity of lime-containing food. Is not the desire for lime shown by these animals analogous to the craving so often exhibited by pregnant women for like substances? It is true the craving does not in every instance take the form of a desire for lime; but nevertheless it is probably only an expression of the same need lacking proper direction.

The system makes cemands for what it needs in a way there is no mistaking. At times we crave acids, and we indulge freely in pickles, acid fruits, etc. At other times sugar or salt is needed, and we eat accordingly.

This is further illustrated by the long pilgrimages made by the buffaloes and antelopes of our Western territory to the salt-licks, in order to satisfy their instinctive demands for salt.

The rapid destruction of teeth during pregnancy, and the therapeutic measures suggested by the so called morbid cravings, are of equal importance to physician and dentist.

To secure for the overtaxed child-bearing woman immunity from much pain, nervous distress, imperfect mastication, and impaired digestion, by the preservation of her teeth, cannot be considered as a trivial matter.

As the sphere of the dentist to day is limited in a great measure to the repair of injuries already sustained by the teeth, we must look to the members of the medical profession to aid us in answering the question. How much in the way of preventing the decay and loss of the teeth from pregnancy can be accomplished by supplying to the system all the lime it needs during the gestative period?—*Phila. Med. Times.*

---

